# Treatment adherence in tyrosinemia type 1 patients

**DOI:** 10.1186/s13023-021-01879-1

**Published:** 2021-06-03

**Authors:** Domingo González-Lamuño, Paula Sánchez-Pintos, Fernando Andrade, María L. Couce, Luís Aldámiz-Echevarría

**Affiliations:** 1 Division of Pediatrics, University Hospital Marqués de Valdecilla, Universidad de Cantabria, and Valdecilla Health Research Institute (IDIVAL), Avda. Valdecilla s/n, 39008 Santander, Spain; 2 Unit of Diagnosis and Treatment of Congenital Metabolic Diseases, Neonatology Service, Department of Pediatrics, University Hospital of Santiago de Compostela, CIBERER, Health Research Institute of Santiago de Compostela (IDIS), MetabERN, A Choupana, s/n, 15706 Santiago de Compostela, A Coruña Spain; 3 Metabolomic Platform, Biocruces Bizkaia Health Research Institute, 48903 Barakaldo, Spain; 4 Unit of Diagnosis and Treatment of Congenital Metabolic Diseases, Neonatology Service, Department of Pediatrics, Hospital Clínico Universitario de Santiago de Compostela, Universidad de Santiago de Compostela, CIBERER, Health Research Institute of Santiago de Compostela (IDIS), MetabERN, A Choupana, s/n, 15706 Santiago de Compostela, A Coruña Spain; 5 CIBERER, Health Research Institute of Santiago de Compostela (IDIS), A Choupana, s/n, 15706 Santiago de Compostela, A Coruña Spain

**Keywords:** Adherence, Diet, Nitisinone, Tyrosinemia type 1

## Abstract

**Background:**

While therapeutic advances have significantly improved the prognosis of patients with hereditary tyrosinemia type 1 (HT1), adherence to dietary and pharmacological treatments is essential for an optimal clinical outcome. Poor treatment adherence is well documented among patients with chronic diseases, but data from HT1 patients are scarce. This study evaluated pharmacological and dietary adherence in HT1 patients both directly, by quantifying blood levels nitisinone (NTBC) levels and metabolic biomarkers of HT1 [tyrosine (Tyr), phenylalanine (Phe), and succinylacetone]; and indirectly, by analyzing NTBC prescriptions from hospital pharmacies and via clinical interviews including the Haynes–Sackett (or self-compliance) test and the adapted Battle test of patient knowledge of the disease.

**Results:**

This observational study analyzed data collected over 4 years from 69 HT1 patients (7 adults and 62 children; age range, 7 months–35 years) who were treated with NTBC and a low-Tyr, low-Phe diet. Adherence to both pharmacological and, in particular, dietary treatment was poor. Annual data showed that NTBC levels were lower than recommended in more than one third of patients, and that initial Tyr levels were high (> 400 µM) in 54.2–64.4% of patients and exceeded 750 µM in 25.8% of them. Remarkably, annual normalization of NTBC levels was observed in 29.4–57.9% of patients for whom serial NTBC determinations were performed. Poor adherence to dietary treatment was more refractory to positive reinforcement: 36.2% of patients in the group who underwent multiple analyses per year maintained high Tyr levels during the entire study period, and, when considering each of the years individually this percentage ranged from 75 to 100% of them. Indirect methods revealed percentages of non-adherent patients of 7.3 and 15.9% (adapted Battle and Haynes tests, respectively).

**Conclusions:**

Despite initially poor adherence to pharmacological and especially dietary treatment among HT1 patients, positive reinforcement at medical consultations resulted in a marked improvement in NTBC levels, indicating the importance of systematic positive reinforcement at medical visits.

## Background

Hereditary tyrosinemia type 1 (OMIM #276700) (HT1) is an inborn error of tyrosine (Tyr) metabolism caused by a deficiency of the enzyme fumarylacetoacetate hydrolase (FAH, EC.3.7.1.2). More than 100 variants in the *FAH* gene, located on the long arm of chromosome 15 (15q23-q25), have been associated with this autosomal recessive entity [1, 2]. Its incidence is estimated at 1 per 100,000 newborns, but is highly influenced by ethnicity-related factors and consanguinity, and is higher in certain geographic regions (e.g. Quebec, Canada) [3]. The deficit in FAH results in the accumulation of toxic metabolites such as maleylacetoacetate, fumarylacetoacetate, and succinylacetone (SA), which are responsible for liver involvement, renal tubulopathy, porphyria-like syndrome, and hepatocellular carcinoma [4, 5].

Diagnostic and therapeutic advances have significantly improved the prognosis of HT1 patients. Treatment is based on the use of nitisinone (2-(2-nitro-4-trifluoro-methylbenzoyl)-1, 3-cyclohexanedione; NTBC), a triketone that blocks the degradation of Tyr early in its metabolic pathway by inhibiting the enzyme 4-hydroxyphenylpyruvate dioxygenase, thereby preventing the production and accumulation of the aforementioned toxic products [6]. An increase in Tyr A levels is a concomitant side effect of NTBC treatment [7]. As such, NTBC therapy should be accompanied by a diet low in Tyr and phenylalanine (Phe), an essential amino acid precursor of Tyr, in order to avoid the deleterious effects of Tyr accumulation, which may underlie recently documented changes in neurotransmitter concentrations in NTBC-treated mice [8].

Usually, it is recommended to maintain plasma Tyr concentrations between ranges 200 and 400 µmol/L up to the age of about 12 years [9]; some authors allow plasma tyrosine concentrations up to 500 µmol/L [10] and even until 600 µmol/L [11]. Phe concentrations is recommended between and 35 and 120 µmol/L [10].

Although evidence indicates that patients who adhere to treatment, even when that treatment is a placebo, have better health outcomes than poorly adherent patients, lack of compliance with therapeutic guidelines is a common phenomenon, especially in patients with chronic diseases [12–14]. Multiple, complex factors underlie this behavior, and include factors related to the patients themselves; to physicians (e.g. communication barriers or ineffective communication of information about adverse effects); and to healthcare systems [15, 16]. Currently, non-compliance with pharmacotherapy and poor adherence to dietary treatment are major healthcare problems that can have serious consequences in terms of quality of life and healthcare system costs, hinder the achievement of positive clinical outcomes, and contribute to treatment failure. In general, due to the low prevalence of inherited metabolic diseases, there are few studies of adherence to diet and pharmacotherapy in this context. One exception is phenylketonuria, studies of which have reported non-adherence to dietary therapy in up to 50% of adult patients [17]. A long-term study found that 18 out of 23 patients with phenylketonuria who initially responded to sapropterin therapy did not continue treatment afterwards [18]. Some adolescents and adults with other metabolic diseases, despite maintaining good metabolic control, may have neuropsychological problems that lead to deficits in executive function and difficulties in planning, organization, and impulse control, which may interfere with their ability to adequately follow medical guidelines [19, 20]. Patients should understand the treatment they are prescribed, and the need for it, in order to be able to organize and take the medication as required, tolerate some of the possible foreseeable side effects, and adhere to their treatment regimen in the long term [21, 22].

HT1 may constitute a useful model with which to study the problem of non-adherence. Studies of adherence to NTBC and dietary therapy in HT1 patients are scarce, but have shown that adherence, especially to dietary guidelines, is generally suboptimal and significantly associated with age, with better adherence observed in young children and poorer adherence in adolescents [7, 23, 24]. Assessment of adherence over a longer period of time, using indirect methods accompanied by a direct quantification [i.e. determination of drug (NTBC) levels in blood], provides a better understanding of true adherence, based on which more realistic improvement strategies can be established. The aim of this study was to evaluate the level of pharmacological and dietary adherence in HT1 patients using direct and indirect measures of non-adherence and to assess the impact of an intervention to positively reinforce treatment adherence.

## Results

Our study population consisted of 69 patients [7 adults and 62 children; 28 (40.6%) women and 41 (59.4%) men] who were seen at 21 different healthcare centers in Spain during the period 2016–2019. The mean and median age was 17 years and 13.2 years, respectively (range 7 months–35 years).

### Pharmacological adherence

#### NTBC levels

Table 1 shows the distribution of patients evaluated by year, as well as corresponding NTBC levels in dried blood samples (DBS) measured in the first determination of the calendar year evaluation and median NTBC levels per year. In the initial determination, lower-than-recommended NTBC levels were detected in more than a third of patients. In patients who underwent multiple annual determinations (11 in 2016; 17 in 2017; 19 in 2018; and 8 in 2019) normalization of NTBC levels within the course of the year was observed in 36.3%, 29.4%, 57.9%, and 25% of patients, respectively.

**Table 1:** Annual evolution of NTBC levels

Year	2016	2017	2018	2019
No. of patients with at least 1 NTBC measurement per year	47	63	63	60
No. patients with > 1 NTBC measurement per year	11	17	19	8
NTBC concentration in first sample of the year (µmol/L)	39	35.9	33.5	35.3
Median NTBC concentration (µmol/L)	39.5	34.3	37.9	34.6
No. of patients (%) with lower than recommended NTBC concentration in first annual determination	16 (34%)	24 (38.1%)	26 (41.3%)	19 (31.6%)
No. of patients (%) in which NTBC concentration normalized within the year	4/11 (36.3%)	5/17 (29.4%)	11/19 (57.9%)	2/8 (25%)

*No* number, *NTBC* nitisinone

Worsening of NTBC levels (i.e. a decrease from the previously normal to lower-than-recommended levels) was observed in 4.3% of the patients evaluated in 2016, 11.5% of those evaluated patients in 2017, and 25% of those evaluated in 2019. In 2018, no changes were observed in any of the patients with initially normal NTBC levels.

The number of determinations per patient did not correlate with better control of NTBC levels in any year during the study period (*p* = 0.636, 0.967, 0.457, and 0.753, respectively). Annual monitoring of NTBC levels neither lead to improvements in adherence among initially non-adherent patients: 76% maintained poor disease control and normalization of NTBC levels was observed in only 6 cases. Remarkably, in these 6 patients achievement of good adherence to NTBC treatment was not accompanied with the normalization of Tyr levels.

### Succinylacetone levels

SA levels were normal, both initially and in subsequent evaluations, in all patients evaluated in 2016 (n = 43), 2018 (n = 56), and 2019 (n = 59). In 2017, SA levels were initially elevated in 2 of 59 patients in the first evaluation, corresponding to the onset period of the disease, but subsequently normalized.

### Dietary adherence

#### Tyrosine levels

Tyr levels measured in DBS ranged from 38.5 to 1368.9 µM in the study population. The percentage of patients with initially higher-than-recommended Tyr levels each year was 55.8% in 2016, 64.4% in 2017; 54.2% in 2018; and 64.3% in 2019. Specifically, 41.9%, of those patients showed Tyr levels in the range of 400–500 µM, 32.2% Tyr levels of 500–750 µM, and 25.8% Tyr levels > 750 µM of patients. In patients with initially higher-than-recommended Tyr levels who underwent several annual Tyr determinations, levels remained elevated in 75%, 92.6%, 100%, and 91.7% of patients in 2016, 2017, 2018, and 2019, respectively. Moreover, 36.2% of these patients maintained poor metabolic control (annual mean Tyr levels > 400 µM) throughout the entire study period. One interesting finding was that 46.1% of patients who had permanently high Tyr levels (n = 26) throughout the entire study period showed good adherence to NTBC treatment (i.e. normal mean NTBC levels) during the entire study period.

When considering patients with poor dietary adherence the age distribution shows that 53.8% were between 12 and 14 years, 15.3% between 15 and 18 years, 23.07% were adults and 3.6% were children < 12 years old.

### Phenylalanine levels

In the study population 43% of patients received Phe supplementation. The percentage of patients with low Phe levels at the first determination was 20.9% in 2016, 32.2% in 2017, 46.5% in 2018, and 27% in 2019. In patients who underwent more than one Phe determination per year, normalization of Phe levels was observed in 42.8% in 2016, 6.2% in 2017, 29.1% in 2018, and 16.6% in 2019. In 10.1% of patients, mean Phe levels remained lower than recommended for the entire study period.

### Correlations between study variables

As shown in Table 2, we observed a significant correlation between NTBC levels and Tyr (*p* = 0.001), between NTBC levels and the Phe/Tyr ratio (*p* = 0.006), and between Tyr and Phe levels (*p* = 0.0001). Evaluation of the correlation between year of analysis (as an independent variable) and both NTBC and Phe (Table 3) revealed a weak correlation.

**Table 2:** Correlations between the variables analyzed

	Tyr	Phe	Phe/Tyr
*NTBC*			
N	217	217	217
Corr	0.247	0.095	− 0.184
R^2^	0.06	0.01	0.03
Sig	**0.0001**	0.165	**0.006**
*Tyrosine*			
N		217	217
Corr		0.439	− 0.408
R^2^		0.19	0.17
Sig		**0.0001**	**0.0001**
*Phenylalanine*			
N			217
Corr			0.245
R^2^			0.06
Sig			**0.0001**

Values in bold are statistically significant

*Corr* Pearson correlation coefficient, *N* number of determinations, *NTBC* nitisinone, *Phe* phenylalanine, *R*^2^ coefficient of determination, *Sig* significance, *Tyr* tyrosine

**Table 3:** Correlation between year of analysis and NTBC and Phe levels

Year	2016	2017	2018	2019
*NTBC*				
Corr	0.23	0.32	0.16	0.43
R^2^	0.05	0.11	0.03	0.19
*Phe*				
Corr	− 0.18	0.26	0.41	0.36
R^2^	0.03	0.07	0.17	0.13

*Corr* Pearson correlation coefficient, *NTBC* nitisinone, *Phe* phenylalanine, *R*^2^ coefficient of determination

### Indirect adherence evaluation methods

The percentage of non-adherent patients as determined by the adapted Battle test was 7.3%: 64 of 69 legal tutors or adult patients responded adequately to the survey questions. In the Haynes test, the percentage of non-adherent patients was 15.9%: 11 of the 69 participants were considered non-compliant.

## Discussion

While adherence to prescribed medicines is recognized as a patient activity essential to maintain self-care and promote well-being, the estimated adherence rate for prescribed medications is about 50% [25]. Although the issue of medication adherence has received greater attention in recent decades, there is a lack of consensus as to the definition and measurement of this variable [26, 27]. Safe and appropriate adherence to medication regimens requires specific knowledge and skills [28]. There are few studies of treatment adherence among patients with rare diseases owing to low prevalence and the difficulty achieving adequate sample sizes. Adherence to dietary and pharmacological treatment is essential in patients with HT1: the majority of patients, if promptly identified and appropriately managed, can live a life free of hepatic or renal disease [10].

Traditionally, treatment outcomes have been attributed to the specific actions of a drug or therapeutic regimen, and consideration of adherence has been limited to its influence on the amount of a specific treatment the patient receives. While direct measures of adherence involving the quantification of a given drug or its metabolite(s) in blood or other bodily fluids represent the gold standard for determining whether a medication has been taken or not, but provide little information about when and how the drug was taken [29]. These biological methods can provide information on adherence, regardless of a patient’s literacy level, but provide little or no information about the patient’s ability to understand, organize, or control their medication use. Pharmacy records are often used to determine whether patients have received their medication on the appropriate date. However, dispensing of a medication does not equate to safe consumption in accordance with the corresponding guidelines.

In the case of HT1, a series of parameters can be used to evaluate different aspects of treatment adherence. These include quantification of biological variables such as levels of NTBC and diet-related metabolites (Tyr, Phe, Phe/Tyr). Because NTBC in Spain is always dispensed from a hospital pharmacy, at each visit patients can be provided with information about the prescribed dose and correct consumption of the medication, and pharmacy records provide a means of verifying that the patient receives the appropriate dose.

This study, which employed a 4-year cohort design, is the first to evaluate adherence to pharmacological and dietary treatment in HT1 patients. Patients who did not adhere to pharmacological and dietary treatment guidelines were identified using direct methods (quantification of NTBC and metabolite concentrations) as well as indirect methods, including evaluation of adherence using the Haynes–Sackett test [30] and the adapted Battle test [31], and monitoring of the filling of NTBC prescriptions at the hospital pharmacy. These measures were accompanied by reinforcement of the knowledge of both patients and primary caregivers about HT1 and the importance of appropriate treatment.

Although nearly 93% of patientes from our cohort have good knowledge of the disease and 84% expressed a good compliance or adherence, objective adherence to pharmacological and, in particular, dietary guidelines was poor. Annual data showed that NTBC levels were lower than recommended in more than a third of patients evaluated, and that Tyr levels measured at the first consultation were high (> 400 µM) in 54.2–64.4% of patients, and very high (> 750 µM) in 25.8% of patients. Remarkably, while normalization of NTBC levels within the course of the year was observed in 29.4–57.9% of patients in which serial NTBC determinations were performed, dietary adherence was more refractory to positive reinforcement. The percentage of NTBC-non-adherent patients in each year in our population (31.6–41.3%) is concordant with published data on non-adherence to treatment in chronic diseases [24] and with previously reported rates of pharmacological and dietary adherence in Spanish HT1 patients [7]. Remarkably, more than one third of the evaluated HT1 patients had persistently lower-than-recommended levels of NTBC, a condition that markedly increases the risk of hepatocellular carcinoma. But quantification of NTBC levels after the positive reinforcement interventions at each clinical visit revealed improvements in 25–58% of patients, depending on the year. Patients who were NTBC-adherent in the initial phases of the project remained adherent throughout the study period: among patients with initially normal NTBC levels, decreases to lower-than-recommended levels were observed in only 10% of them in subsequent measurements. Nonetheless, a substantial percentage of non-NTBC-adherent patients remained non-adherent despite intervention measures aimed at improving treatment adherence. This observation may explain the lack of association between NTBC levels and the number of determinations per year. A detectable or increased concentration of SA in blood spots, plasma, or urine is considered a sensitive indicator of suboptimal NTBC treatment and a cause for treatment adjustment [32]. In our study population elevated SA levels were observed only in 2 patients in their first determination, corresponding to clinical onset.

Dietary adherence was unsatisfactory in more than a half of all HT1 patients (54.2–64.4%), as reflected by persistently higher than recommended annual Tyr levels, and high-risk Tyr levels (> 750 µM) were detected in 25.8% of patients. In contrast to NTBC adherence, no improvement in dietary adherence or metabolic control was observed in initially non-diet- adherent patients.

Although elevated Tyr levels could be explained by the administration of NTBC, as reflected by the slight positive correlation observed between Phe and NTBC levels (R^2^, 0.06). NTBC inhibits activity of the enzyme 4-hydroxyphenylpyruvate dioxygenase, which acts upstream in the Phe degradation pathway. Therefore, the increase in Tyr levels seems to be of little relevance, considering the low value of R^2^. The fact that a considerable percentage of HT1 patients (46.1%) had persistently high Tyr levels despite good NTBC adherence highlights the importance of ensuring dietary, and not just pharmacological, adherence in order to achieve adequate metabolic control.

In HT1 management Phe supplementation is commonly indicated in patients with persistently low Phe plasma levels, as deficiency of this amino acid can lead to neurological damage. In our study population, a remarkably high percentage of patients had low plasma Phe levels.

One of the factors that could help explain the poor treatment adherence observed in our study population is the age of the participating patients (median age, 13.2 years). Deterioration of treatment adherence in teenagers with chronic diseases is well recognized phenomenon [33], and is influenced by physical, social, and emotional changes that occur during adolescence. Adolescents commonly have trouble adequately adhering to strict treatment regimens that they feel constitute an unnecessary burden and set them apart from their peers [34]. The age distribution of non-dietary adherents in our population, with 69.1% of them between 12 and 18 years of age, clearly shows that adolescence is a key stage to adherence maintenance. HT1 patients tend to report poorer adherence to both medication and diet than their caregivers do [23], possibly due to a greater awareness of caregivers about the importance of treatment to ensure an optimal disease course. In fact, patients with caregivers who perceive the disease as a serious disorder have better treatment adherence than those with few HT1 symptoms. These differences between caregivers and patients highlight the importance of a proper disease information as a key factor to continued adherence. Moreover, during consultations with their physician, adolescents often adopt a non-participatory approach, giving all the attention to their parents. Decreasing interaction with parents and increasing it with adolescents could improve future adherence. Patients’ perceptions and beliefs are also important predictors of non-adherence. Greater involvement in their disease management and in consultations during the pre-adolescent period can have a positive impact on future adherence by improving their understanding of the disease and its treatment, potentially leading to greater stability and attenuating the effects of negative influences at this stages of life. Self-reporting methods are an effective means of assessing patient adherence. These methods are fast and inexpensive, and allow identification of potential psychosocial barriers to adherence [29]. However, self-reporting methods can overestimate compliance and may be affected by social factors and recall bias, limiting their usefulness [35]. Moreover, these methods may not be appropriate for patients with low literacy levels, for whom reading and completing forms can be particularly difficult. Another aspect to keep in mind is the importance of patient empowerment in treatment adherence [36]. At the individual level, empowerment encompasses the patient’s autonomy and their perceived capacity to effectively cooperate and share disease control with their physician. Further research will be needed to determine the appropriate level of control that should be allocated to the patient and the physician, respectively, in different contexts (i.e. in different conditions or stages of the disease).

The results of this observational study indicate that treatment success can be improved by enhancing adherence. One limitation of our study is that our analysis did not consider social, emotional, and cultural factors, which may significantly influence treatment adherence in rare diseases with high morbidity (e.g. HT1). Moreover, there is a lack of evidence-based guidelines regarding optimal target blood NTBC concentrations, although accepted values are generally in the range of 30–60 µM. Key strengths of this study include the optimal sample size (despite HT1 being a rare disease) and its mixed-method, observational cohort design.

## Conclusions

Our results reveal poor adherence to pharmacological and, in particular, dietary treatment in Spanish patients with HT1, in line with findings in patients with other chronic diseases. In our study population positive reinforcement via clinical visits resulted in a notable improvement in blood NTBC levels, underscoring the importance of systematic reinforcement during medical consultations with HT1 patients. Improving adherence could lead to changes that directly or indirectly influence patient outcomes. Further research will be needed to develop multifactorial strategies to improve adherence, barriers to which are both complex and varied.

## Methods

### Study design and population

This was on observational study of HT1 patients who were being treated in Spanish centers with NTBC and a low-Tyr, low-Phe diet during the period January 1, 2016–December 31, 2019. Individual patient data were provided by 21 centers after informed consent was provided by patients or, in the case of minors or disabled patients, their parents/guardians. Patients in treatment for less than 6 months and those who had received a liver transplant were excluded. All participating centers sent DBS to the Metabolomic Platform of the Biocruces Bizkaia Health Research Institute (Barakaldo, Spain) for quantification of NTBC, SA, Tyr, and Phe levels. Participating centers were recommended to send 3 samples per year. The minimum information required to accept patient samples was age, sex, and hematocrit levels (%). In cases in which more than one sample was provided per year, mean and median concentrations of NTBC, Tyr, Phe, and SA were evaluated in the first sample received each calendar year.

Reference intervals for biochemical parameters were as follows: NTBC, 30–60 µM; Tyr, 34–202 µM; Phe, 28–100 µM; SA, ≤ 0–1.5 µM. The cut-off point for adequate Tyr control was set at < 400 µM and recommended Phe levels (35–120 μm/L) were established according the Spanish recommendations [37].

### Methodology for measuring treatment adherence

The following methods were used to measure adherence:

- Direct methods: evaluation of pharmacological adherence by measuring the concentration in DBS of NTBC and of SA and Tyr (metabolic biomarkers of HT1); evaluation of dietary adherence by measuring of Tyr and Phe levels and determining the Phe:Tyr ratio.
- Indirect methods: based on the clinical interview to patients or parents/legal tutors in case of pediatric patientsHaynes-Sackett (or self-compliance) test [30] and the adapted Battle test (test of patient knowledge about the disease), both of which were conducted at the first medical consultation of the calendar year [31].

The adapted Battle test consisted of the following three questions: (1) Is HT1 a lifelong disease?; (2) Can HT1 be controlled with diet and treatment?; (3) Indicate two or more organs that can be affected in HT1 patients. An incorrect response to any of these 3 questions was considered indicative of a non-adherent patient.

The Haynes test consists of asking the patient about their level of compliance with treatment. First, to create an environment of trust the following statement is introduced “most patients have difficulty taking all their tablets”. Then, the patient is asked whether they also have difficulty taking their treatment. If the patient answers in the affirmative, they are considered non-compliant. However, a negative response does not rule out that the possibility that the patient is non-adherent for other reasons. For this reason, the following follow-up questions are posed: *How do you take them?; How often do you complete your medication? Always, many times, sometimes, never?* Finally, a third question is posed to evaluate the patient’s response: *Many patients have difficulties in continuing their treatment, why not tell me how you are doing?* If the patient’s response to any of the questions posed indicates difficulties complying with treatment guidelines, they are considered non-adherent.

### Positive reinforcement strategy to improve adherence

Positive reinforcement, which consisted of remembering the therapeutic plan and its impact on its evolutionary prognosis in clinical visits, was systematically provided to improve therapeutic adherence.

### Reagents and solutions

Pure NTBC (Orfadin®) was provided by the pharmaceutical company SOBI (Swedish Orphan Biovitrum, Stockholm, Sweden). Mesotrione, an insecticide structurally similar to NTBC and used as an internal standard, was supplied by Sigma-Aldrich (Madrid, Spain). Formic acid was purchased from Merck (Madrid, Spain) and Teknokroma (Barcelona, Spain) provided methanol and water. All reagents used were of the purest quality available (performed by high performance liquid chromatograph- HPLC).

The chromatography system used consisted of an Agilent 1100 Series equipped with an Agilent Zorbax C8 column (150 × 4.6 mm, 5 μm). The tandem mass spectrometer (Agilent 6410B triple quad) uses electroionization as the ionization source. The drug and internal standard were detected in multiple reaction monitoring (MRM) mode, selecting the most intense and selective transitions for each molecule.

### Analytical methodology

#### Blood samples

Blood samples were acquired by venipuncture of the antecubital vein after overnight fasting and collected in EDTA tubes. A Pasteur pipette was used to transfer blood drops onto Whatman 903 paper cards, which were dried horizontally at room temperature for approximately 4 h before storage or shipment of the sample.

#### NTBC analysis

NTBC concentrations were quantified as previously described by Prieto et al. [38]. Briefly, a 3-mm diameter punch was removed from the Whatman card and placed in a plate to which mesotrione in methanol was added for compound extraction. The plate was agitated at room temperature for 20 min, and the solution subsequently injected into the liquid chromatograph-tandem mass spectrometer.

#### Amino acid analysis

Quantification of Tyr, Phe, and SA levels was carried out using the Chromsystems kit (Gräfelfing, Germany) for the determination of acylcarnitines and amino acids in DBS by tandem mass spectrometry. Briefly, compounds were extracted from a 3-mm DBS punch following the addition of internal standard solution for amino acids and SA. The supernatant was completely dried before derivatization, and then evaporated before reconstitution and injection into the tandem mass spectrometry system.

### Statistical analyses

The variables analyzed in this study were recorded in an anonymized database created for this purpose and processed using the free statistical analysis program R-commander. A descriptive statistical analysis of each of the variables in the database study was performed. Qualitative variables were compared using the Chi-squared test or Fisher test. The normality of quantitative variables was assessed using the Kolmogorov–Smirnov or Shapiro–Wilk test, depending on the size of the sample. Analyses of pairs of quantitative variables were performed using the Pearson or, in cases of non-normally distributed data, the Spearman correlation coefficient. Comparisons of quantitative and qualitative variables were performed using the Student’s t-test for independent samples. Statistical significance was set at *p* < 0.05.

## Data Availability

The datasets used and/or analyzed in this current study are available from the corresponding author upon reasonable request.

## Abbreviations

DBS: Dried blood sample
FAH: Fumarylacetoacetate hydrolase
HT1: Hereditary tyrosinemia type 1
NTBC: Nitisinone
PHE: Phenylalanine
SA: Succinylacetone
TYR: Tyrosine
